# Characteristics and mechanism of local scour reduction around spur dike using the collar in clear water

**DOI:** 10.1038/s41598-024-63131-7

**Published:** 2024-05-29

**Authors:** Hongliang Qi, Jie Wang, Wen Zou, Wenjun Luo, Weiping Tian, Jiachun Li

**Affiliations:** 1 Key Laboratory for Special Area Highway Engineering of Ministry of Education, School of Highway, Chang’an University, Xi’an, 710064 China; 2 Hexagon Software Technology Co., Ltd., Qingdao, 266000 China

**Keywords:** Spur dike, Local scour reduction, Collar, Model test, Numerical simulation, Hydrology, Natural hazards

## Abstract

To reduce the local scour around the spur dike, the U-shaped collar is proposed in this study. The influence of the collar's length, width, and porosity on the local scour reduction in clear water is studied by model tests and numerical simulations. Experimental studies show that the collar has a significant effect on reducing the local scour. The location of the maximum scour depth of the spur dike moves downstream. The width of the collar has the greatest impact on the reduction effect among the three selected factors, followed by the porosity and the length. Local scour reduction efficiency of the collar can reach 56.9%. Based on the regression analysis of the range and variety, a formula for predicting the reduction effect around the spur dike is put forward, and the deviation between the values by formula and that in experiments are within ± 4%. The characteristics of the flow field around the spur dike under constant conditions with a collar are studied via numerical simulation. The numerical simulation results show that compared to the case without collar, the flow velocities around the spur dike in cases with permeable collar and solid collar reduced by 45% and 25%, respectively, and the shear stresses reduced by 20% and 28.6%, respectively. The results of this study can provide a reference for local scour reduction using the solid collar or collar made of permeable materials such as gabions.

## Introduction

Spur dike has been widely used as a current deflector in river training, bank protection, and many other fields. However, spur dikes can also contract the river channels and change the flow structure which causes the vortices around the spur dikes. The flow structure is a complex, three-dimensional turbulence. As a result, the local scour around the spur dike threatens the lifetime of the spur dike and the objects which are protected. A large number of studies have been carried out to understand the mechanism of local scour, the structure of water flow, the vortex characteristics, and the scour reduction measures of spur dike. Meaningful results have been achieved^1^.

Radan^2^ studied the effect of the submergence percentage of the T-shaped spur dike on the scour and flow pattern in a 90° bend. The distance between the maximum scour depth and spur dike situation was fixed by changing the submergence ratio, which is 0.6 times the flow height, at the channel inlet. Mohammad^3^ studied the flow and scour patterns in a 90° bend when installing two T-shaped spur dikes. The secondary flow energy occurs at the upstream of the first spur dike and the maximum sedimentation occurs at the end of the inner bank. Mehraein^4^ investigated the scour hole dimensions around submerged and emerged spur dikes in a 90° bend in mean and turbulent flow fields. The bed shear stress estimated by TKE and the scour process has a direct relation. Maryam^5^ compared variations of the mean flow pattern in a 180° bend with a variety of lengths of T-shaped spur dike. The results showed that when the spur dike was placed at the bend apex, the flow strength near that range increased by approximately 2.5 times. Li^6^ studied the influence of permeability on the flow characteristics and riverbed deformation around the spur dike. The results showed that the separation angle and scouring depth of the solid spur dike were greater than that of the permeable spur dike. Liu^7^ analyzed the effects of different spur dike spacing on water level, flow velocity, and length of the reflux zone. The results showed that the local scouring could be effectively improved by adjusting the spacing and number of spur dikes. Wen^8^ studied the influence of the spur dike angle on the flow characteristics of the bend through model tests. The results showed that when the spur dike angle was 135°, the water level changed the least and the flow pattern was the best. Zheng^9^ recommended the design parameters of the permeable spur dike and concluded that the length of the spur dike should be selected according to the contraction rate of the river. Han^10^ used flume tests to measure the three-dimensional velocity components around triangular and rectangular spur dike. The results showed that the maximum scour depth around the triangular spur dike was smaller than that of the rectangular spur dike. The flow fields of the spur dikes interact with each other, and the scour characteristics of the spur dikes were related to the spacing of the spur dikes.

As computational technology has developed by leaps and bounds, Computational Fluid Dynamics (CFD) has been used widely in the study of local scour topography of structures. Vaghefi^11^ investigated the flow field around a T-shaped spur dike located in a 90° bend. The results showed a significant effect of the spur dike on the secondary flow patterns. Vaghefi^12^ studied the effect of the submergence ratio on the flow pattern around a short T-head spur dike in a mild bend with a rigid bed using a numerical model. Mohammad^13^ investigated the effect of a T-shaped spur dike on flow separation in a 90° bend using the SSllM model. Zhang^14^ conducted a three-dimensional numerical simulation of the flow structure near the spur dike and found that under the same crossing section, the number of spur dike stages increased, and the reflux zone of the downstream spur dike increased. Li^15^ studied the influence of incoming flow conditions on the local scour of the stepped spur dike through numerical simulation. The results showed that the time required for scour to reach equilibrium was mainly affected by incoming flow conditions. A multiphase flow model was adopted by Ning^16^ to simulate the local scour topography of the spur dike and revealed the changing rule of the flow field and the characteristics of the scour pit around the spur dike. The simulation results of Fluent by Hu^17^ showed that under the same angle of the spur dike, the average velocity near the spur dike decreases with the increase of water depth. The numerical simulation results were compared with the flume tests, and the conclusions were consistent.

Based on the mechanism of local scour, the reduction measures can be divided into two categories: the active protection measures and the passive protection measures^18^. Soft mattress protection^19^ is formed by arranging geotextiles with certain dimensions and placing geotechnical weights on the soft mattress to form an integrated structure, which forms good structural stability and can be applied to different terrains. It is found by Kumcu^20^ that placing a collar on or below the bed of a bridge abutment can reduce the maximum depth of scour significantly than that of the bridge abutment without the collar in experimental studies. The effect of the apron on the local scour of spur dike was studied by Melville^21^. The results showed that the local scour depth would increase with the increase in the width of the revetment. The effect of different materials on the apron was compared, and the stone apron showed the best effect. Ding^22^ concluded that a stepped spur dike could change the flow path of water and reduce the flow velocity to a certain extent, which helps to maintain the stability of the riverbed by model test. Zheng^9^ investigated the flow characteristics and the scour mechanism of the permeable gabion spur dike via numerical simulation and proposed the values of the design parameter of the permeable gabion spur dike. Jiang^23^ derived the influencing factors affecting the local scour of spur dike based on years of experience in the destruction of spur dike and conducted an experimental study on the stability of the riprap spur dike. It shows that the reinforcement of the foundation is often adopted to reduce the scour capacity of the flow, as well as to maintain the safety and stability of the foundation of the spur dike.

Much research has been carried out by scholars all over the world, and there are a lot of achievements in the mechanism of local scour and local scour reduction measures of spur dike, such as the riprap, gabions, and foundation platforms. However, there are obvious shortages in these measures. For example, the location of the riprap is vulnerable to the flow and needs regular replenishment. The surface of the gabion is vulnerable to wear and tear of external forces, and the connection of the grid loosens easily. The cost of the foundation platform is huge, but the protective effect beyond the scope of the design is poor. A new local scour reduction measure is proposed in this paper, the collar. The shielding effect of the collar is used to block the impact of the falling water on the riverbed around the spur dike, and the purpose of protecting the spur dike is achieved by inhibiting the formation of the horseshoe vortex. The collar can also overcome the shortcomings of riprap, gabions, and foundation platforms. The influence of the length, width, and porosity of the collar on the local scour reduction and the reduction mechanism in the clear water is analyzed by combining the model tests and numerical simulation. The results of the study can provide a reference for mitigating the local scour of new and existing spur dike.

## Methods

### Factor analysis

Many factors can affect the depth of local scour at spur dike, including spur dike factors (narrowed rate of rivers, deflect angle, and permeability characteristics), channel and flow characteristics (sediment characteristics, flow velocity, and water depth), and characteristics of scour reduction measures.

Liu^24^ found that a short spur dike was more effective for bank protection. The narrowed rate of the river should be less than 0.33. Zheng^9^ found that the maximum local scour depth around the spur dike increased with the increase of the length of the spur dike, and the narrowed rate of the river should be between 0.15 and 0.25. Tian^25^ concluded that a positive pick spur dike was more appropriate from many experimental tests and on-site projects. It is proposed by Zhang^26^ that the maximum local scour depth of the spur dike decreases with the increase of the permeability, and the decrease tends to stabilize gradually.

The channel and flow characteristics are closely related to the local scour of the spur dike. Flume tests were applied by Zhao^27^ to study the effect of bed sand particle size on local scour. The results showed that when the bed sand particle size is small, it is easy to be carried away by the flow, and the scour depth increases. However, when the bed sand particle size is smaller than a certain value, the scour depth decreases with the decrease in particle size. Gao^28^ found that the maximum scour depth decreases with the increase of bed sand inhomogeneity coefficient and the bottom of the scour pit will be "roughened". Dou^29^ found that when the flow velocity is less than the incipient velocity of the sediment, the local scour depth keeps increasing as the flow velocity increases. Fang^30^ found that the incipient velocity of bed sand decreases when water depth increases, while the local scour depth increases with the increase in water depth. Raudkivi^31^ concluded from indoor tests that the maximum local scour depth initially increases with the flow depth. When the flow depth exceeds a certain height, the scour depth will not change any longer.

Studies on the local scour reduction around bridge piers with collars are fruitful. Fang^32^ found that when the diameter of the collar is 3.0 times the diameter of the pier, the protection effect is the best. Jahangirzadeh^33^ found that when the diameter of the collar is 3.0–3.5 times the diameter of the pier, it reaches the most cost-effective range. However, if the size of the collar keeps growing, the protective effect does not increase. Most of the studies focused on solid collars and did not take the effect of permeability on the efficiency of collar scour reduction into consideration. Studies on the local scour reduction around spur dikes with collars are rarely reported. The local scour mechanism of the spur dike and pier is very close, the parameters of the collar based on the existing results are selected in this study. Based on protection measures such as riprap and gabions, the effect of permeability on the efficiency of local scour reduction is investigated as well.

### Experimental system

**Figure 1:**
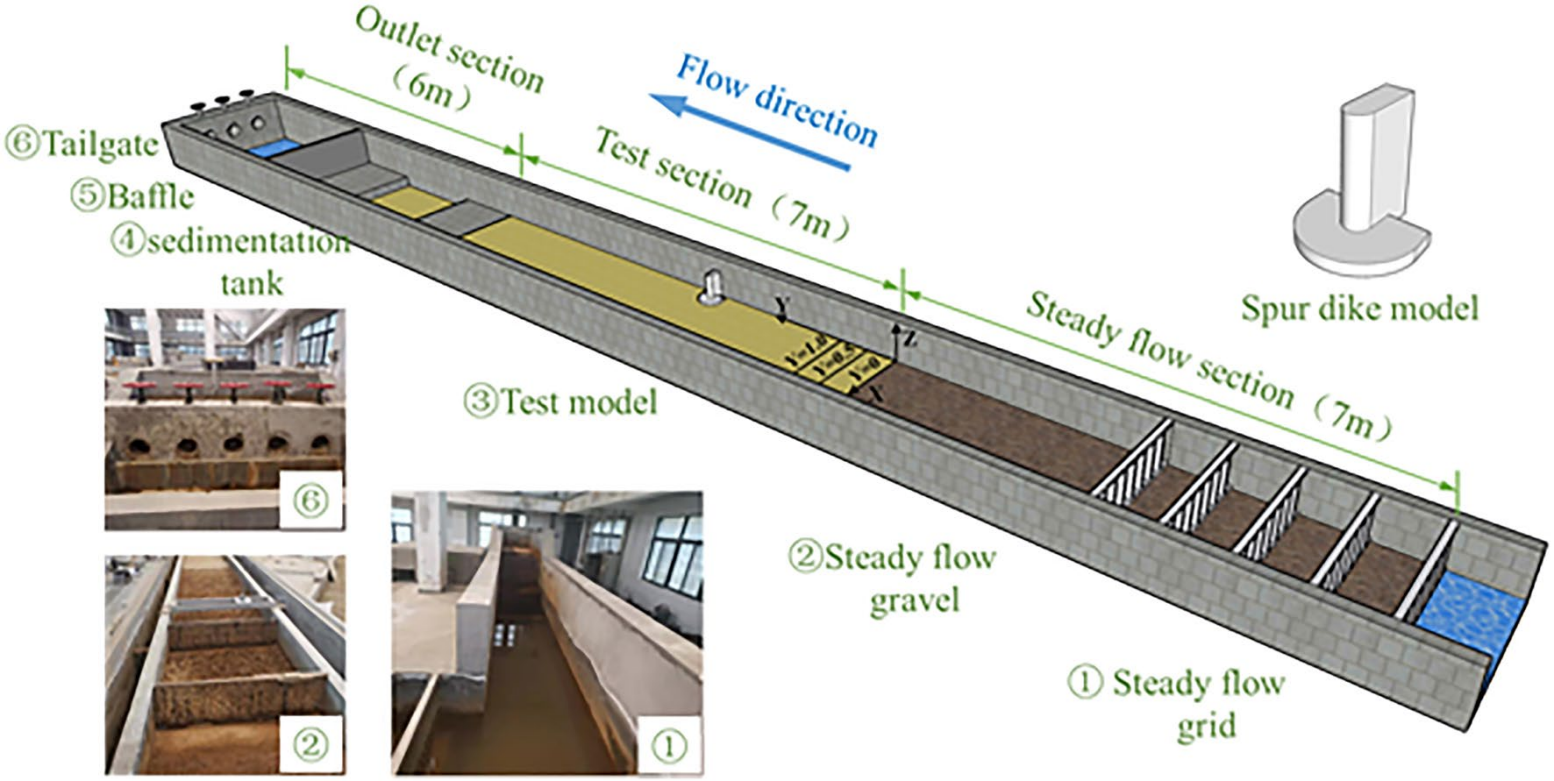
The layout of the test system. SketchUp 2020. https://adobe.sytskji.cn/m5/6537c9d14d88b31f28553644?sdclkid=AsFpbLjpALei15AG&bd_vid=2255898298393140016.

The experiments were carried out in the Key Laboratory for Special Area Highway Engineering of the Ministry of Education at Chang’an University. The flume was 20.0 m long, 1.6 m wide, and 0.7 m high. The side walls and bottom of the flume were plastered with cement. The experimental flume was equipped with a water supply and circulation system, as shown in Fig. 1. To make sure the water flew into the test section uniformly, a 7.0 m long fixed bed section was set at the inlet of the flume with 5 straighter. The test section was in the middle of the test flume with a length of 7.0 m, the bottom of which was covered by sediment with a thickness of 0.15 m. Reynolds and Froude numbers of the flow condition are 37,519 and 0.25, respectively. The spur dike is a cuboid with a semi-cylindrical end. It was located on the right side of the test section. The deflection angle was 90°. The bottom of the spur dike was equipped with a fixed support for stability. The water depth was 0.2 m. The scour depth and scour feature maps were measured with a laser distance meter.

### Sediment and flow velocity

**Figure 2:**
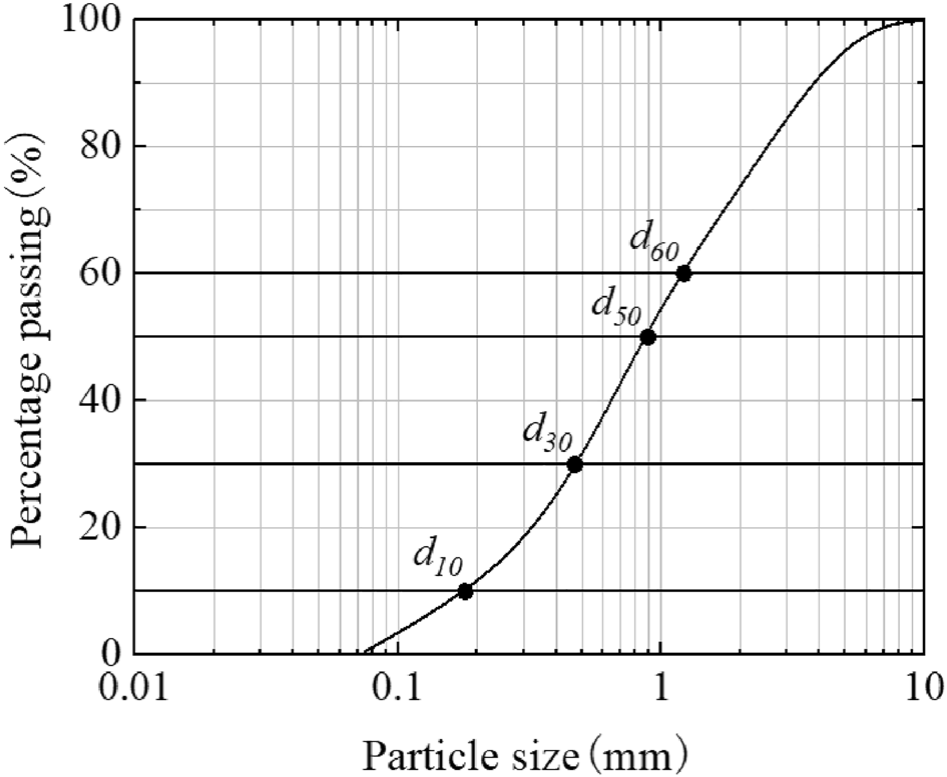
Gradation curve of the sediment.

The influence of the length, width, and porosity of the collar on scour reduction characteristics were the targets of this research. So, the sediment was uniform. The median particle size *d*_*50*_ was 0.89 mm, the uniformity coefficient *C*_*u*_ was 7.17, and the curvature coefficient *C*_*c*_ was 1.03. The gradation of the sediment could meet the requirements of the test. The gradation curve of the sediment is shown in Fig. 2.

**Figure 3:**
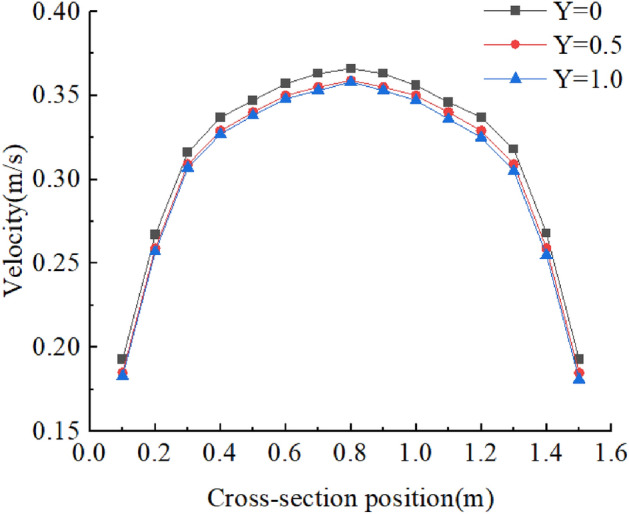
Cross-sectional flow velocity.

It shows the flow velocity distribution of cross-sections at different locations in Fig. 3. The flow velocity distribution is close to reality, and the average flow velocities of each cross-section were 0.312 m/s, 0.309 m/s, and 0.306 m/s, respectively, which could be regarded as a uniform flow. The incipient velocity of the sediment was 0.349 m/s which was the result of Zhang Ruijin’s formula, and is shown below:

1 $${v}_{0}={\left(\frac{h}{d}\right)}^{0.14}{\left(29d+0.000000605\frac{10+h}{{d}^{0.72}}\right)}^{0.5}$$

where *v*_0_ is incipient velocity, *h* is the depth of the water, and *d* is the average size of sediment. The flow velocity of the test was less than the incipient velocity of sediment, so this study was in clear water conditions.

### Experimental setup

**Figure 4:**
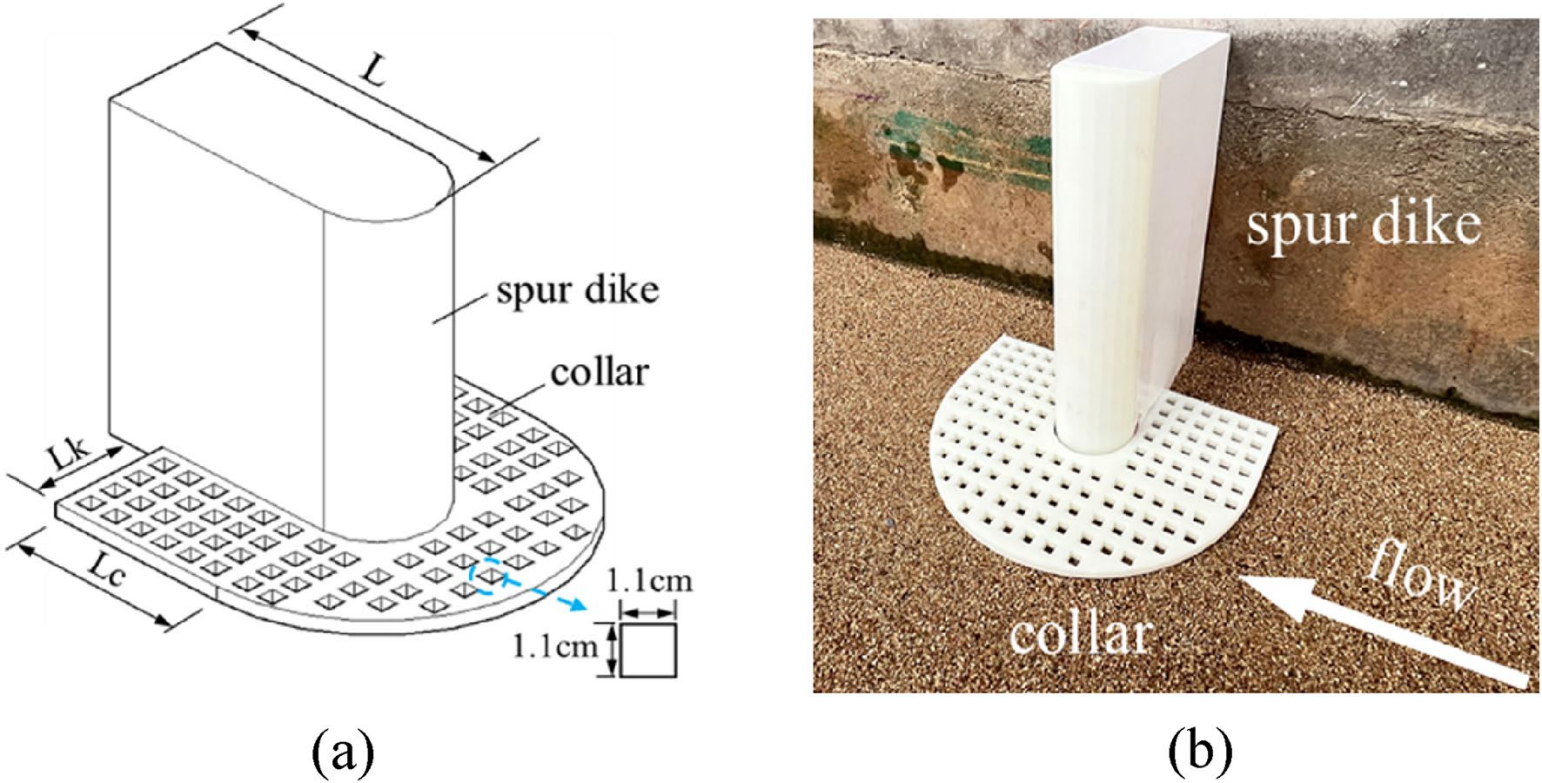
Layout of the collar (Lc/L = 0.4, Lk/L = 0.6, γ = 25%): (**a**) Experimental setup. (**b**) Experimental preparation.

The experimental model diagram is shown in Fig. 4. The spur dike was composed of a rectangle and a semicircle at every section. It formed a "U" shape and was nested around the spur dike. The collar was installed on the river bed. L was the length of the spur dike, *L*_*C*_ was the length of the collar, *L*_*K*_ was the width of the collar, and *γ* was the porosity of the collar. In this study, the dimensionless length *L*_*C*_*/L*, the dimensionless width *L*_*K*_*/L,* and the porosity* γ* were selected as the main factors of the collar in the condition of constant flow and water level. For *L*_*C*_*/L* and *L*_*K*_*/L*, there were five levels and four levels of porosity, as shown in Table 1. The test in which the spur dike is not protected was added for comparative analysis.

**Table 1:** Factors and levels of the test.

Level	Factors		
	*L*_*C*_*/L*	*L*_*K*_*/L*	*γ*
1	0	0.2	0
2	0.2	0.3	0.25
3	0.4	0.4	0.5
4	0.6	0.5	0.7
5	0.8	0.6	–

An interface meter was fixed near the spur dike to measure the development of the local scour depth in the test. Before the test starts, the sand bed of the test section should be leveled to the design height. Then a laser distance meter was used to fine-level the sediment surface of the test section by the measuring grid (the grid size of the area around the spur dike was 1.0 cm × 1.0 cm, and the grid size of the rest of the area was 2.0 cm × 2.0 cm). The deviation was within 0.1 mm.

## Results and discussions

### Local scour around spur dike without the collar

**Figure 5:**
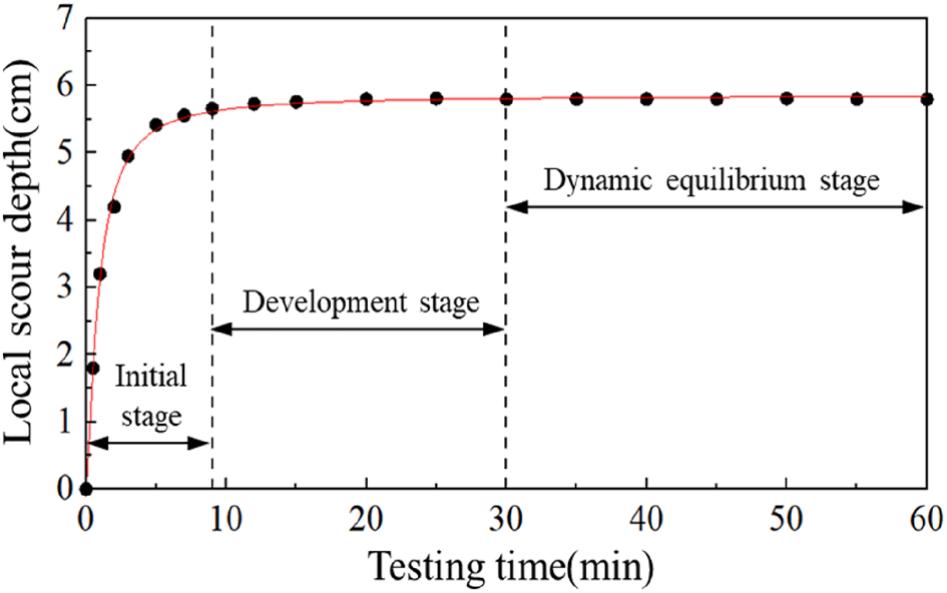
Time-dependent scour depths without a collar.

The relationship between local scour depth and time of the spur dike without a collar is shown in Fig. 5. The local scour depth increased rapidly within the first 10 min and slowed down gradually in 30 min, then reached stable in 40 min, which indicated that the local scour had reached a dynamic equilibrium state. The characteristics of the local scour development of the spur dike observed were consistent with the existing research results. Zhao^34^ found that when the local scour depth reaches 95% of the equilibrium scour depth, the scour can be considered approximately to reach equilibrium. The scour depth became stable within 60 min in this study. Therefore, to make sure that the scouring process was in the dynamic equilibrium stage, the scouring time of subsequent tests was 120 min in the study.

**Figure 6:**
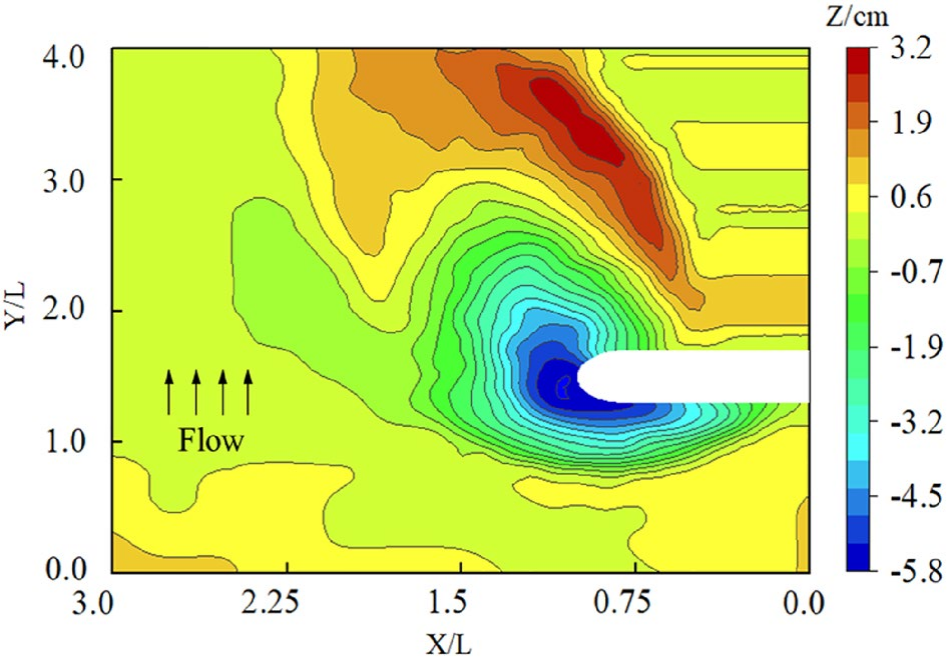
Local scouring around a spur dike without a collar.

The local scour topography of the spur dike without a collar is shown in Fig. 6. The maximum local scour depth was 5.8 cm. A significant accumulation of sediment was generated at about 0.5*L* behind the spur dike. It was because the upstream flow was blocked and contracted by the spur dike, and the structure of the flow was changed drastically. The combined action of descending currents and the horseshoe vortex carried sediment away from the area around the spur dike, which created the local scour pits. The flow velocity recovered gradually behind the spur dike, and the vortex disintegrated gradually, which caused the depositing of sediment gradually.

### Reduction efficiency of collar

**Figure 7:**
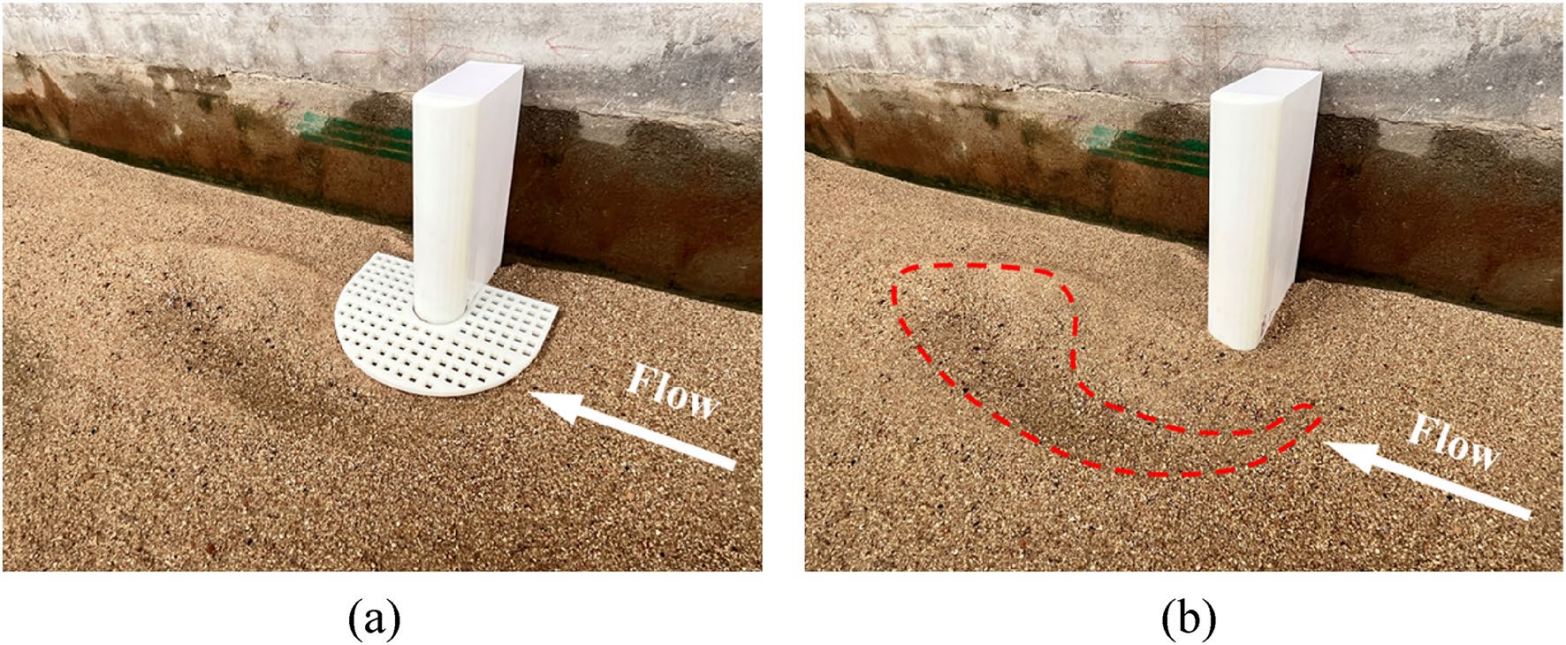
Local scour around the spur dike with collar (Lc/L = 0.4, Lk/L = 0.6, γ = 25%): (**a**) Local scour topography. (**b**) Topographic map of local scour.

**Figure 8:**
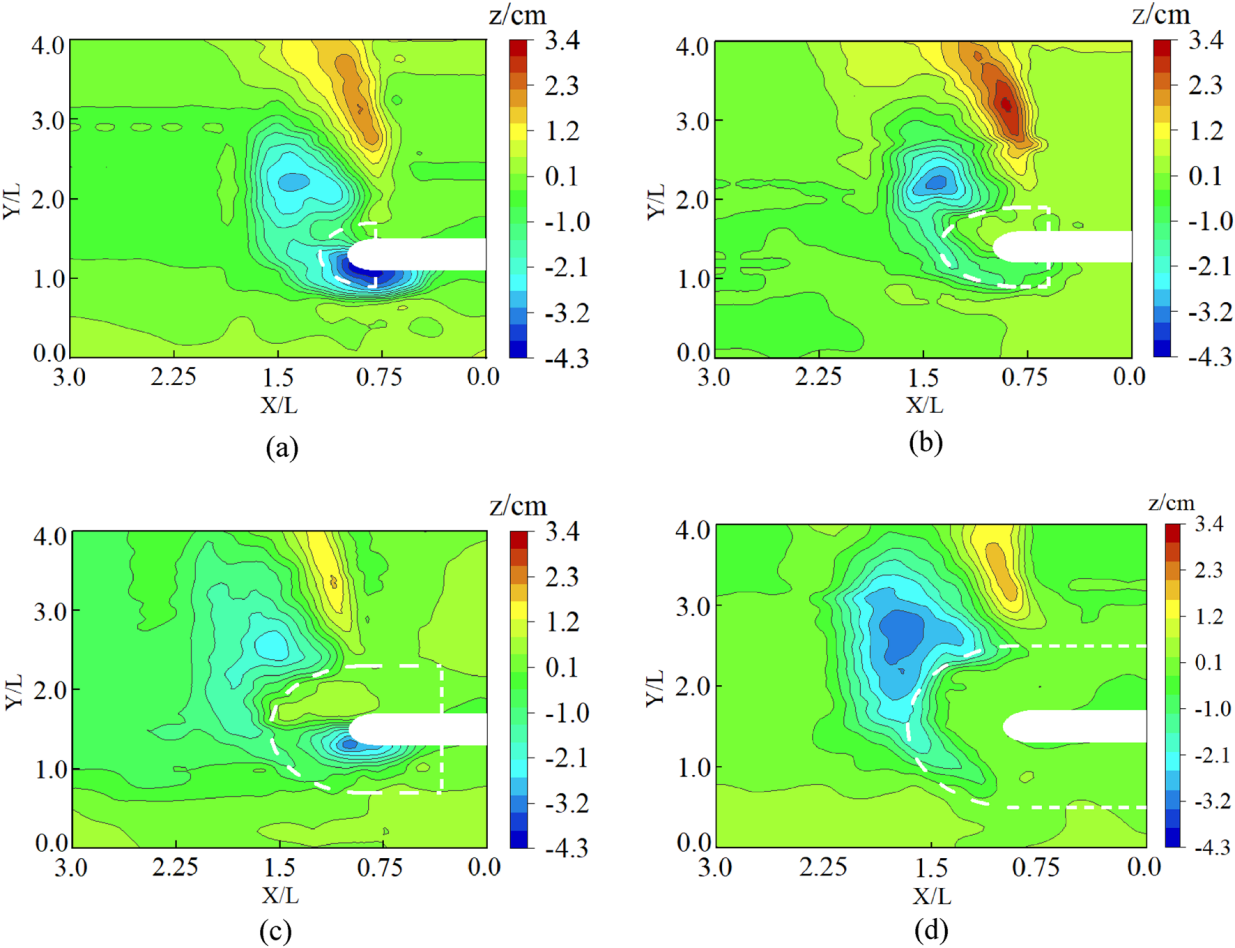
Contour maps around the spur dike with collar: (**a**) Group 1 (L_C_/L = 0, L_K_/L = 0.2, γ = 0). (**b**) Group 7 (L_C_/L = 0.2, L_K_/L = 0.3, γ = 0.25). (**c**) Group 19 (L_C_/L = 0.6, L_K_/L = 0.5, γ = 0.7). (**d**) Group 25 (L_C_/L = 0.8, L_K_/L = 0.6, γ = 0.25).

The local scour topography and contour maps of the spur dike with the collar are shown in Figs. 7 and 8. The results showed that the local scour depth around the spur dike decreased significantly with different collar parameters. When the size of the collar was increased or the porosity was decreased, the reduction effect of the collar increased gradually. The reason was that the collar changed the flow structure around the spur dike and prevented the submerged currents and horseshoe eddies. Therefore, the local scour near the spur dike was reduced. On the other hand, the collar weakened the eddies and changed the direction of the downstream flow. The location of the maximum scour depth around the spur dike was moved downstream as a sequence.

To analyze and compare the reduction effect of the collar quantitatively, the percent of the local scour reduction, *R* (%), is introduced, and expressed as:

2 $$R=\left({d}_{se}-{d}_{sec}\right)\times 100/{d}_{se}$$

where *d*_*se*_ is the maximum local scour depth around the spur dike without a collar, and *d*_*sec*_ is the maximum local scour depth around the spur dike with a collar. The maximum local scour depth and *R* (%) of each test are shown in Table 2.

**Table 2:** The maximum local scour depth and R (%) of each test.

Test no.	Factor			Maximum local scour depth around the spur dike (cm)	*R (%)*
	*L*_*C*_*/L*	*L*_*K*_*/L*	*γ*		
1	0	0.2	0	4.3	25.9
2	0	0.3	0.5	3.8	34.5
3	0	0.4	0.25	3.6	37.9
4	0	0.5	0.25	3.4	41.4
5	0	0.6	0.7	3.7	36.2
6	0.2	0.2	0.25	3.5	39.7
7	0.2	0.3	0.25	3.3	43.1
8	0.2	0.4	0.7	3.5	39.7
9	0.2	0.5	0	3.1	46.6
10	0.2	0.6	0.5	3.3	43.1
11	0.4	0.2	0.7	4.1	29.3
12	0.4	0.3	0	2.7	53.4
13	0.4	0.4	0.5	3.6	37.9
14	0.4	0.5	0.25	3.2	44.8
15	0.4	0.6	0.25	3.2	44.8
16	0.6	0.2	0.5	3.9	32.8
17	0.6	0.3	0.25	3.5	39.7
18	0.6	0.4	0.25	3.2	44.8
19	0.6	0.5	0.7	3.6	37.9
20	0.6	0.6	0	2.9	50
21	0.8	0.2	0.25	3.5	39.7
22	0.8	0.3	0.7	4.1	29.3
23	0.8	0.4	0	3.2	44.8
24	0.8	0.5	0.5	3.4	41.4
25	0.8	0.6	0.25	3.3	43.1
26	Without collar			5.8	–

**Figure 9:**
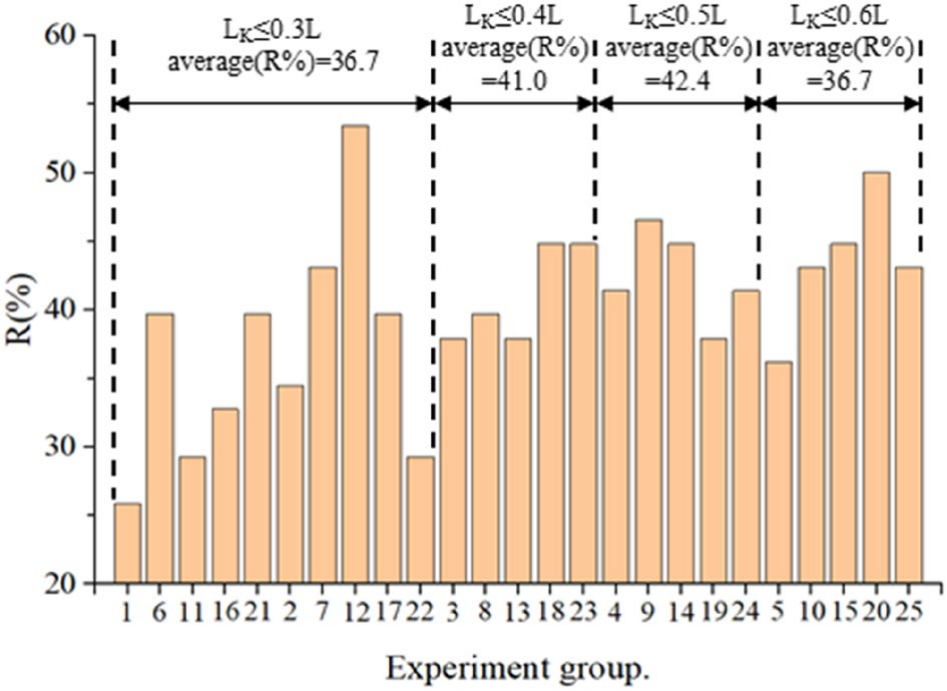
R (%) of the collar in tests.

The percent of the local scour reduction *R* (%) of the different collars in experimental tests are shown in Fig. 9.

According to the variation of the width of the collar *L*_*K*_ and *R* (%), the collars can be divided into four groups, *L*_*K*_ ≤ 0.3*L*, *L*_*K*_ = 0.4*L*, *L*_*K*_ = 0.5*L*, and *L*_*K*_ = 0.6*L*. When *L*_*K*_ ≤ 0.3*L*, *R* (%) of different collars was relatively small but fluctuated greatly, the average value of *R* (%) was 36.7 and the standard deviation was 8.1; When *L*_*K*_ = 0.6*L*, *R* (%) of different collars was relatively large and had similar values, the average value of *R* (%) was 43.4 and the standard deviation was 4.9; When *L*_*K*_ = 0.4*L* or 0.5*L*, *R* (%) of each collar was significantly improved compared with *L*_*K*_ ≤ 0.3*L* but fluctuated more, the average value of *R* (%) were 41.0 and 42.4, the standard deviation were 3.2 and 3.0.

### Main effects analysis of maximum scour depth

For each factor, the average value of the experimental results at the same level can be calculated by Eq. (3):

3 $${K}_{ij}=\frac{\sum {Y}_{ij}}{n}, \quad i=\text{1,2}, \ldots,\text{m}$$

where *m* is the total number of factors, *n* is the total number of levels, ${Y}_{ij}$ is the experimental result with factor *i* at level* j*.

*R*_*i*_ represents the difference between the maximum and minimum values of ${K}_{ij}$ and it can be calculated by Eq. (4):

4 $${R}_{i}=\text{max}\left({K}_{i1}, \ldots,{K}_{in}\right)-\text{min}\left({K}_{i1}, \ldots ,{K}_{in}\right)$$

${R}_{i}$ reflects the fluctuation of the experimental results of the factor *i* at different levels. The influence of different factors on the experimental results can be obtained by sorting the *R* of each factor. A greater value of *R*_*i*_ means the effect of this factor on the experimental results was more significant. The influence of the factor at different levels on the results could be obtained by analyzing the different levels of the specified factor.

Table 3 shows the average value of each level of the factor. According to Table 3, the width of the collar ranked first among the three factors, followed by the porosity of the collar and the length of the collar, which indicates that the reduction effect of the maximum scour depth around the spur dike was strongly influenced by the width of the collar. However, the average value of each level of the factor was close, which means it was the result of a combination of the three factors.

**Table 3:** The average value of each level of the factor (cm).

Factor	*L*_*C*_	*L*_*K*_	*γ*
Factor level	Average maximum scour depth		
*K1*	3.76	3.86	3.24
*K2*	3.34	3.48	3.32
*K3*	3.36	3.42	3.6
*K4*	3.42	3.34	3.8
*K5*	3.5	3.28	–
R	0.42	0.58	0.56
Rank	3	1	2

**Figure 10:**
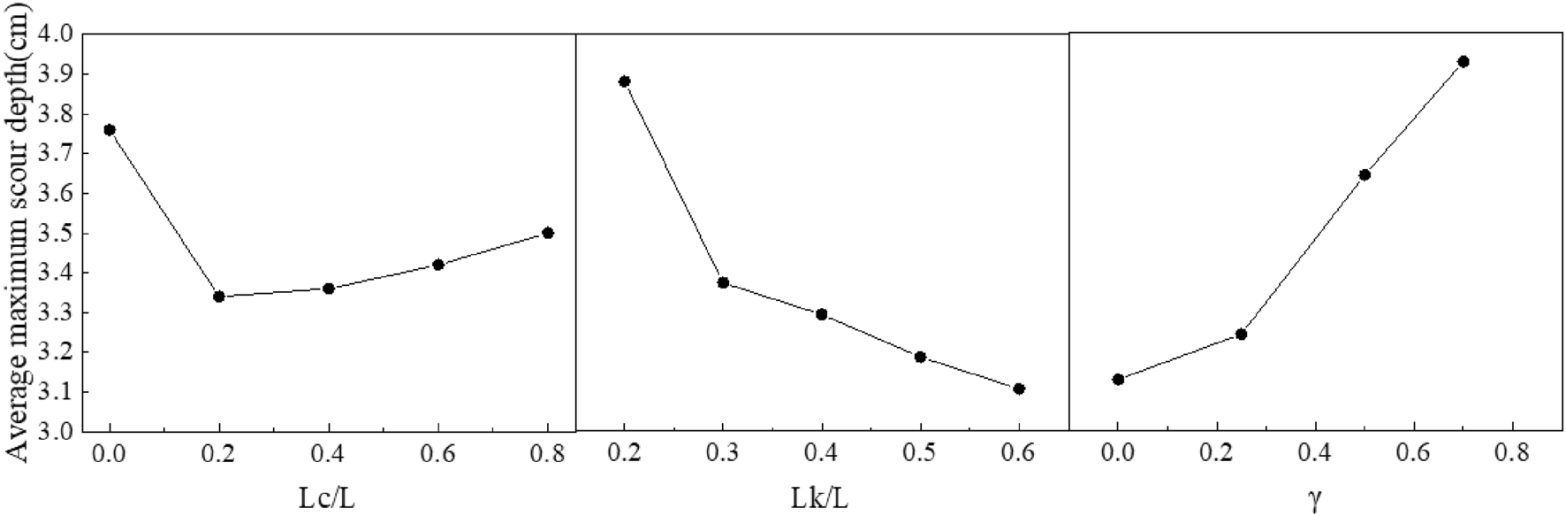
Main effect plots of the maximum scour depth around the spur dike.

Figure 10 shows the effects of different levels of the same factor on the maximum local scour depth. The maximum scour depth decreased rapidly as the width of the collar increased, and decreased rapidly with the increase in the length of the collar when *L*_*c*_/*L* ≤ 0.2, then increased gradually as the length of the collar increased. The maximum scour depth increased rapidly when the porosity of the collar increased.

### Analysis of variance for maximum scour depth

ANOVA is a standard statistical technique that can be used to calculate the variability from data. It can be adopted to evaluate whether or not the factors investigated in the experiment had a significant effect on the experimental results objectively.

The amount of all squared deviations, *S*_*T*_, the sum of squared deviations for individual factors, *S*_*A*_, and the sum of squared deviations of errors, *S*_*E*_, need to be calculated. *S*_*T*_ represents the deviation of the experimental data from the average and can be calculated by the following equation.

5 $${S}_{T}=\sum_{i=1}^{n}{\left({Y}_{i}-\overline{Y }\right)}^{2}$$

where *n* is the overall number of experiments, ${Y}_{i}$ is the result of the *i*th experiment, $\overline{Y }$ is the average value of ${Y}_{i}$.

The sum of squared deviations for individual factors can be calculated by Eq. (6).

6 $${S}_{A}={\sum_{k=1}^{L}\frac{1}{{n}_{k}}\left[\sum_{i=1}^{{n}_{k}}\left({A}_{ik}-{Y}_{0}\right)\right]}^{2}-\frac{{T}^{2}}{n}$$

where ${n}_{k}$ is the number of experimental tests when the factor level is taken as *k*, *L* is the total number of levels, ${A}_{ik}$ is the value of the experimental result when the factor level is s *k*, ${Y}_{0}$ is the imaginative value of the experimental result, *T* is the sum of the deviations of the experimental results from the imaginative value.

The sum of squared deviations of errors can be calculated by Eq. (7).

7 $${S}_{E}={S}_{T}-{S}_{A}$$

As *S*_*A*_ and *S*_*E*_ are independent, the ratio of their mean squares (*MS*) comes up with the F-distribution, and the F-value can be calculated by the following Eq. (8).

8 $${F}_{j}=\frac{M{S}_{j}}{M{S}_{e}}\sim F\left({f}_{j},{f}_{e}\right)$$

When ${F}_{j}>{F}_{1-\alpha }\left({f}_{j},{f}_{e}\right)$, the factor can be significant at the significance level α.

The ANOVA results of this study are shown in Table 4. The F-values at 95% and 90% confidence intervals are 3.26 and 2.48, respectively. It can be seen that the width and porosity of the collar had a significant effect on the local scour depth reduction around the spur dike at a 95% confidence interval. The three design parameters of the collar had a significant effect on reducing the local scour around the spur dike at a 90% confidence interval. The order of the effect of the factors on the reduction of the maximum scour depth around the spur dike was the width of the collar > the porosity of the collar > and the length of the collar, which was consistent with the conclusion of the main effect analysis.

**Table 4:** Analysis of variance for maximum scour depth.

Source	*S*	*DOF*	*MS*	*F*
*L*_*C*_	0.582	4	0.145	2.544
*L*_*K*_	1.038	4	0.259	4.544
*γ*	1.018	4	0.254	4.456
Error	0.689	12	0.057	
Sum	3.327	24		

### Prediction of R (%)

The regression analysis was used to predict the local scour reduction of the collar around the spur dike. Based on the experimental data of the R (%), the formula for the prediction of the R (%) was fitted. Kumar^35^ found that the formula for prediction calculation was only related to the structural parameters of the collar. It is agreed in this paper. The design parameters of the collar were considered ideal. The functional relationship between the reduction efficiency R of the collar and the length of the collar, *L*_*C*_*/L*, the width of the collar, *L*_*K*_*/L,* and the porosity of the collar, *γ*, is as follows:

9 $$R=f\left(\frac{{L}_{C}}{L},\frac{{L}_{K}}{L},\gamma \right)$$

10 $$R={C}_{0}{\left(\frac{{L}_{C}}{L}\right) }^{{C}_{1}} {\left(\frac{{L}_{K}}{L}\right) }^{{C}_{2}} {\left(\gamma \right) }^{{C}_{3}}$$

where *C*_*0*_, *C*_*1*_, *C*_*2*_, *C*_*3*_ are constant coefficients. The polynomial form of Eq. (11) is shown below:

11 $$\mathit{ln}(R)= \mathit{ln}({C}_{0})+{C}_{1}\mathit{ln} \left(\frac{{L}_{C}}{L}\right)+{C}_{2}\mathit{ln} \left(\frac{{L}_{K}}{L}\right)+{C}_{3}\mathit{ln}(\gamma )$$

Based on the regression analysis and the experimental data, the values of constant coefficients *C*_*0*_, *C*_*1*_, *C*_*2*_, and *C*_*3*_ are in Table 5.

**Table 5:** Constant coefficients in Eq. (11).

Coefficients	*C*_*0*_	*C*_*1*_	*C*_*2*_	*C*_*3*_	*R*^*2*^
Value	37.5555	− 0.0614	0.1943	− 0.1986	0.924

The *R*^*2*^ of the fitted formula is 0.924, which could be used to prove that the correlation between the fitted formula and the experimental data was good. Therefore, the formula for the local scour reduction calculated around the spur dike by using a collar is as follows:

12 $$R=37.5555{\left(\frac{{L}_{C}}{L}\right) }^{ -0.0614} {\left(\frac{{L}_{K}}{L}\right) }^{0.1943} {\left(\gamma \right) }^{-0.1986} { L}_{C}>0, {L}_{K}>0, \gamma \ge 0$$

**Figure 11:**
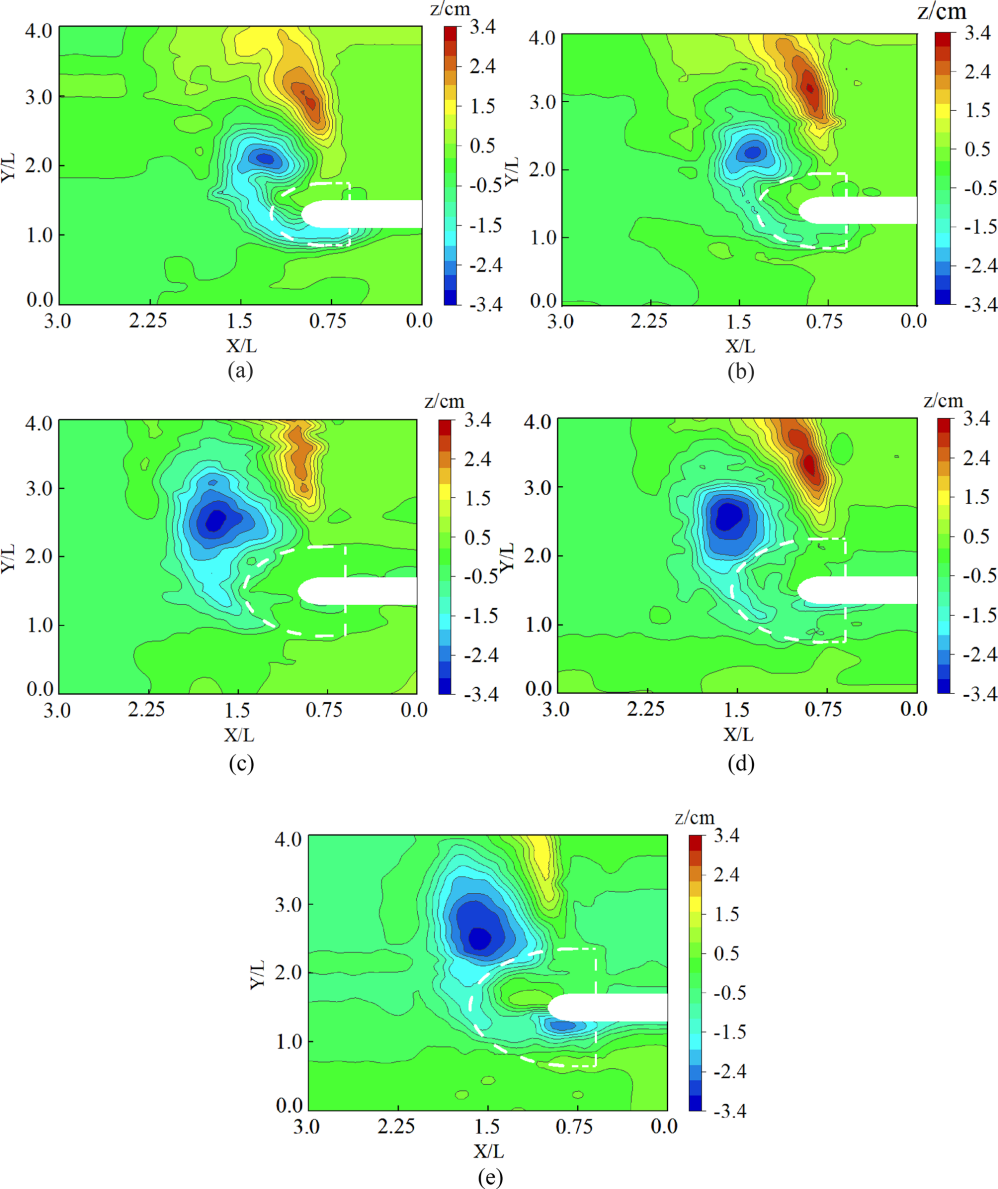
Contour maps around the spur dike with collar: (**a**) (L_C_/L = 0.2, L_K_/L = 0.25, γ = 0.1). (**b**) (L_C_/L = 0.2, L_K_/L = 0.35, γ = 0.2). (**c**) (L_C_/L = 0.2, L_K_/L = 0.45, γ = 0.3). (**d**) (L_C_/L = 0.2, L_K_/L = 0.55, γ = 0.4). (**e**) (L_C_/L = 0.2, L_K_/L = 0.65, γ = 0.6).

To further verify the accuracy of formula (12), 5 more experimental tests were carried out in this study. The parameters of collars in each test and the *R* (%) are in Table 6. The reliability of the formula was verified by comparing the measured value of R (%) with the predicted value. The local scour topography of the 5 experimental tests is shown in Fig. 11.

**Table 6:** Test results and calculated values of *R* (%).

Test No	Factor			Maximum scour depth around the spur dike (cm)	*R (%)*
	*L*_*C*_*/L*	*L*_*K*_*/L*	*γ*		
1	0.2	0.25	0.1	3.0	48.1
2	0.2	0.35	0.2	3.0	47.8
3	0.2	0.45	0.3	3.2	44.8
4	0.2	0.55	0.4	3.3	43.1
5	0.2	0.65	0.6	3.4	41.4

**Figure 12:**
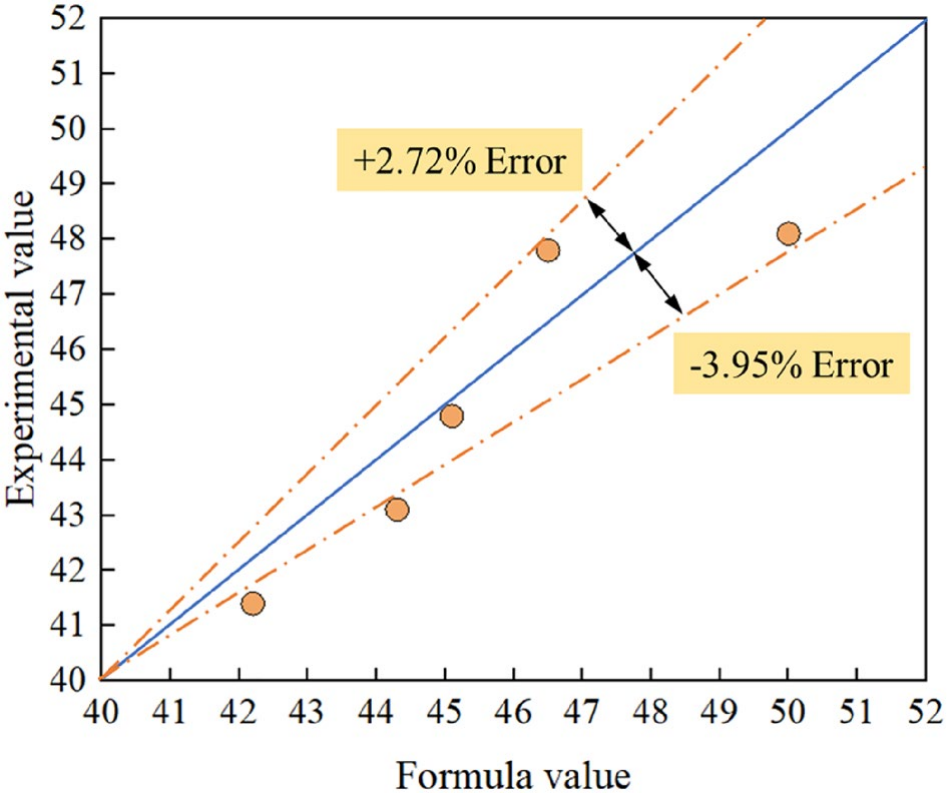
Formula verification.

The verification results are shown in Fig. 12. The deviations between the calculated values and those in the experiment were within ± 4%, which means the formulas proposed in this paper are accurate.

### Numerical simulation

The CFD (Computational Fluid Dynamics) software Fluent was used to investigate the mechanism of local scour reduction around the spur dike with collar, mainly targeted to the characteristics of the flow field and shear stress near the surface of the sediment. So far, the numerical studies of flow fields have been carried out under fixed bed conditions^36^. Therefore, the numerical simulations in this study followed the same conditions.

### Numerical theory

The turbulence models are widely used in Fluent, such as the standard k-ε model, RNG k-ε model, Spalart–Allmaras model, Large Eddy Simulation model, etc. The standard k-ε turbulence model was selected in this study:

Continuity equation:

13 $$\frac{\partial {u}_{i}}{\partial {x}_{i}}=0$$

Turbulent energy k equation:

14 $$\frac{d{u}_{i}}{dt}={f}_{i}-\frac{\partial p}{\partial {x}_{i}}+\frac{\partial }{\partial {x}_{j}}\left[\left(v+{v}_{t}\right)\left(\frac{\partial {u}_{i}}{\partial {x}_{j}}+\frac{\partial {u}_{j}}{\partial {x}_{i}}\right)\right]$$

Dissipation rate ε equation

15 $$\frac{d\varepsilon }{dt}=\frac{\partial }{\partial {x}_{i}}\left[\left(v+\frac{{v}_{t}}{{\sigma }_{\varepsilon }}\right)\frac{\partial \varepsilon }{\partial {x}_{i}}\right]+{C}_{1\varepsilon }\frac{\varepsilon }{k}{p}_{k}-{C}_{2\varepsilon }\frac{{\varepsilon }^{2}}{k}$$

where *t* is time, *u*_*i*_ is the velocity component, *x*_*i*_ is the coordinate component, *v* is the kinematic viscosity coefficient, *p* is the corrected pressure, *f*_*i*_ is the mass force, *v*_*t*_ = *c*_*u*_*k*^*2*^*/ε* is the turbulent viscosity coefficient,* c*_*u*_ = 0.09, *σ*_*k*_ = 1.0, *σ*_*ε*_ = 1.33, *c*_*1ε*_ = 1.44, *c*_*2ε*_ = 1.42, *G*_*k*_ is the turbulent kinetic energy generation term caused by the average velocity gradient, *α*_*w*_ is the volume fraction of water.

### Simulation verification

Before the numerical simulation, the local scour tests of Ning^37^ were used to verify the numerical method. The test flume was 4.0 m long, 0.4 m wide and 0.15 m high. The groin was 0.1 m long and 0.01 m wide and was located on the right bank. The spur dike was the same size and was also located on the right bank. The inlet flow rate was 9.62 L/s, and both sides and the bottom were Smooth Walls. The outlet and top were Pressure, and the inlet was the Velocity Inlet.

**Figure 13:**
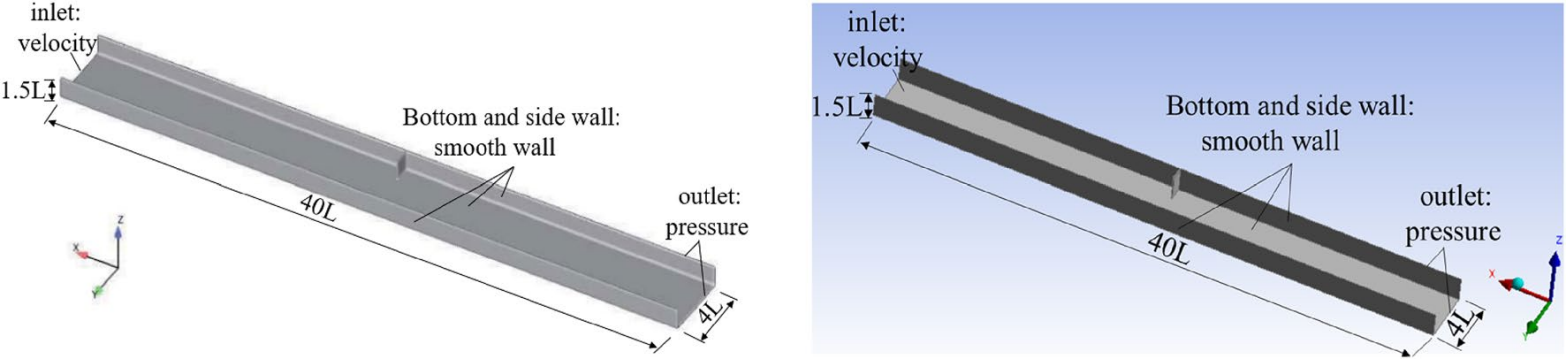
Three-dimensional numerical models. Workbench 2022 R1. https://www.ansys.com/zh-cn/products/ansys-workbench.

**Figure 14:**
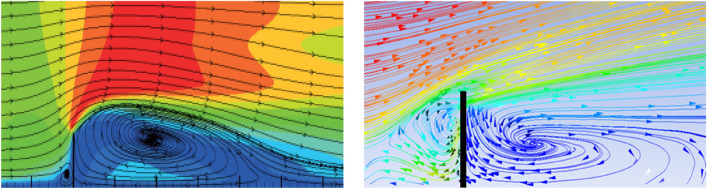
Comparison of flow characteristics.

Figure 13 shows the three-dimensional numerical model based on the scour test of Ning^37^. The comparison results between the simulation of water flow characteristics and Ning Jian's study are shown in Fig. 14. The results showed that when the water flow was blocked by the spur dike, the backflow area in front of the spur dike was generated. The velocity of water flow decreased significantly. After the water bypassed the spur dike, a large reflux area was formed downstream.

**Figure 15:**
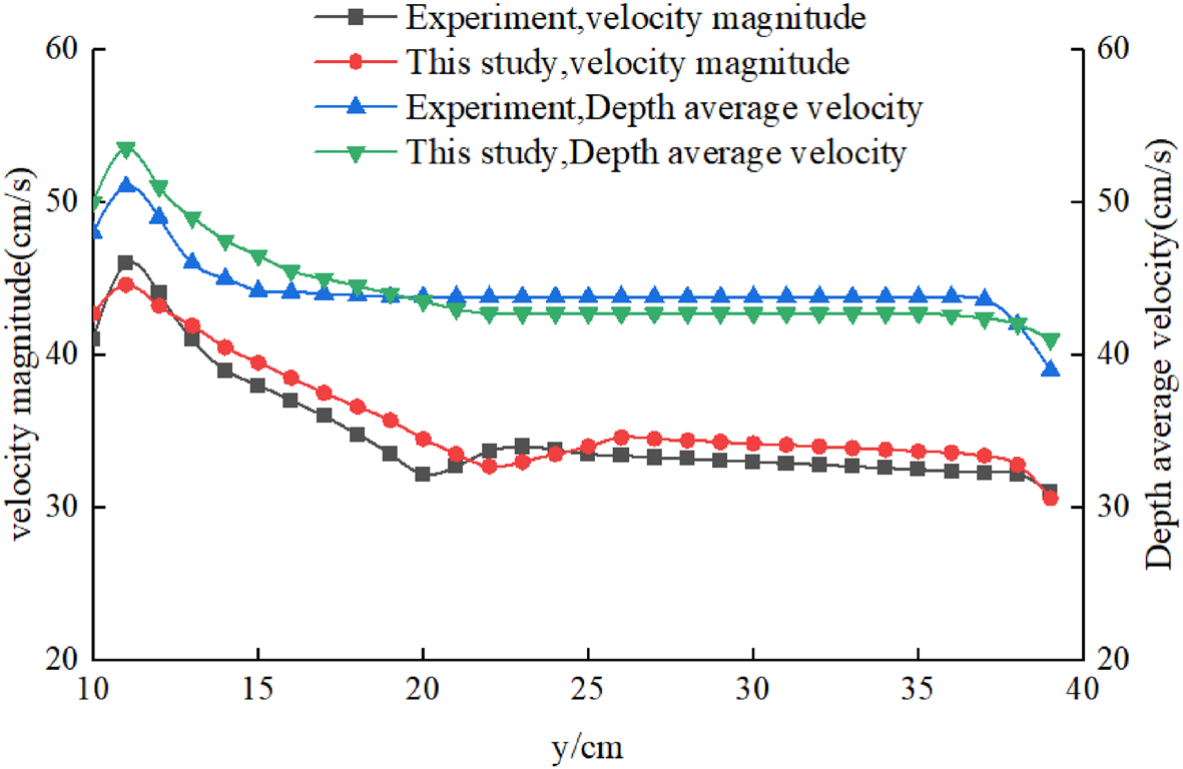
Velocity distribution at the spur dike cross-section near the bed.

Figure 15 shows the distribution of velocity magnitude and depth average velocity at the spur dike cross-section near the bed. The results showed that the water flow structure was the same. In summary, the simulation results were in good agreement with the experimental results. The numeric method was correct and feasible.

### Model setup

**Figure 16:**
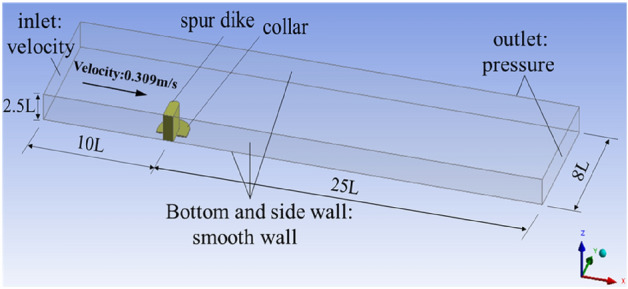
Three-dimensional numerical models. Workbench 2022 R1. https://www.ansys.com/zh-cn/products/ansys-workbench.

A three-dimensional numerical model with the same size as the model test was built, as shown in Fig. 16. The length of the numerical model was 35.0*L*, the width was 8.0*L* and the height was 2.5*L*. The spur dike was located at x = 0 on the right side of the channel. The distance between the spur dike and the inlet was 10.0*L* and the distance between the spur dike and the outlet was 25.0*L*. Numerical simulation CFD calculation parameters are shown in Table 7.

**Table 7:** Numerical simulation calculation parameters.

Boundary condition	Boundary type
Inlet	Velocity inlet (0.309 m/s)
Outlet	Pressure (pressure = 0)
Top	Pressure (pressure = 0)
Bottom	Smooth wall
Side-walls	Smooth wall

**Figure 17:**
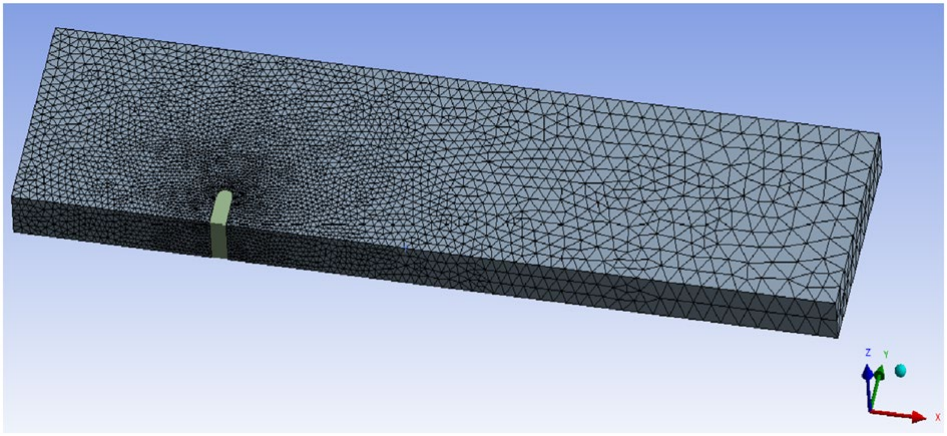
Meshing of the model. Workbench 2022 R1. https://www.ansys.com/zh-cn/products/ansys-workbench.

According to the results of Jiang^38^ of the time step and grid independence of CFD results, when the ratio of mesh size to model width was less than 0.048, the mesh size did not affect the calculation any longer. The model grid was tetrahedral, with a uniform size of 0.02 m and a maximum size of 0.05 m. The mesh around the spur dike was encrypted locally to improve the mesh quality. The grid size was 0.01 m, and the growth rate was 1.1. The grid contained 8,084,623 cells in total. It shows the mesh of the model in Fig. 17. The ratio of mesh size to model width was 0.025 in this study. This met the requirement of grid independence. The case numbers of numerical simulation in this study were: No.26, No.19 (L_C_/L = 0.6, L_K_/L = 0.5, γ = 0.7), and test with the solid collar (L_C_/L = 0.6, L_K_/L = 0.5, γ = 0).

### Velocity of the flow field

**Figure 18:**
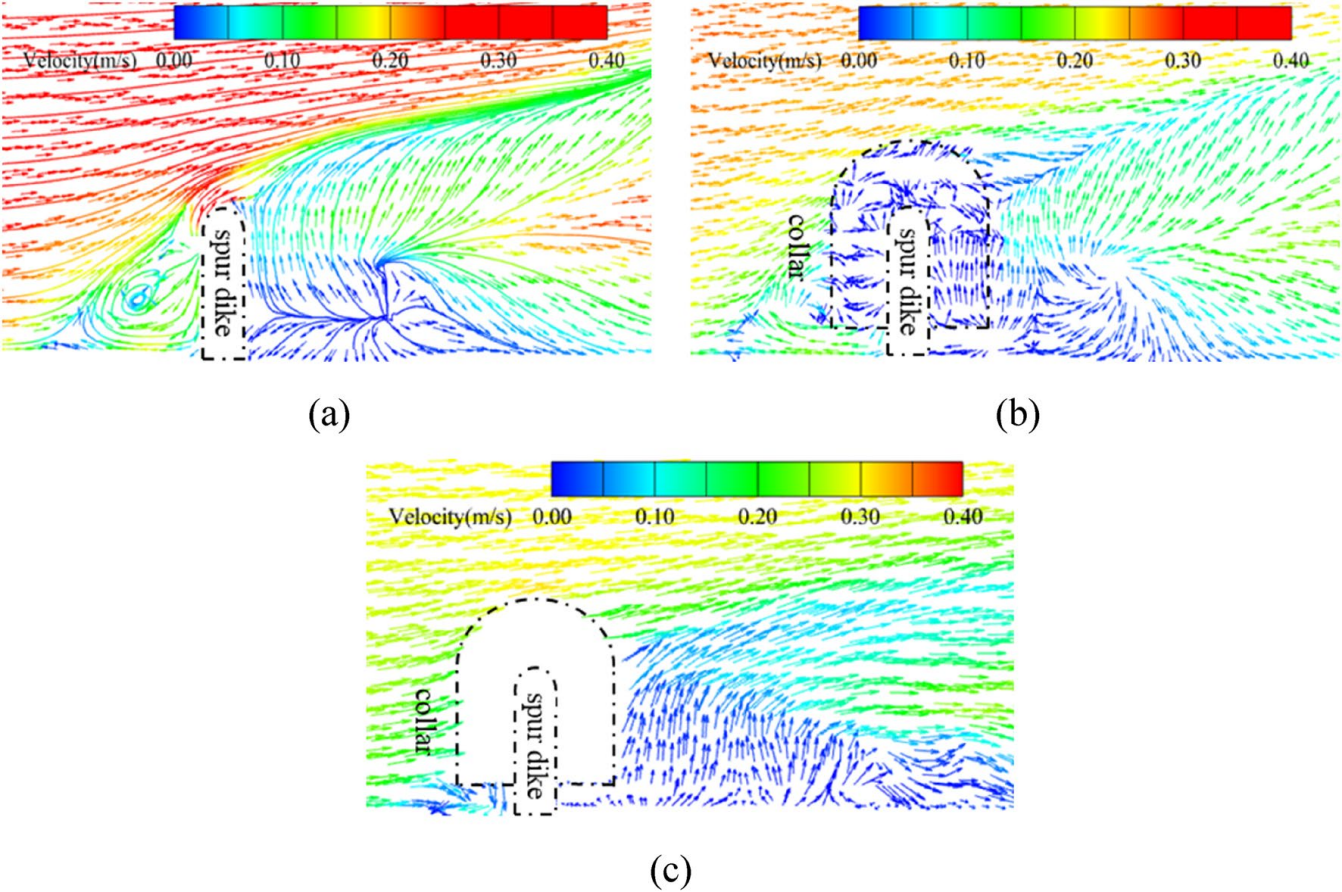
Velocity vectors around the spur dike (z = 0.005 m). (**a**) No.26 (without collar). (**b**) No.19 group (L_C_/L = 0.6, L_K_/L = 0.5, γ = 0.7). (**c**) Test with the solid collar (L_C_/L = 0.6, L_K_/L = 0.5, γ = 0).

Figure 18 shows the velocity vector diagram of the horizontal section at 0.005 m above the bottom of the riverbed in cases No.26 (without collar), No.19 (L_C_/L = 0.6, L_K_/L = 0.5, γ = 0.7), and the test with the solid collar (L_C_/L = 0.6, L_K_/L = 0.5, γ = 0).

Figure 18a shows that when the spur dike was unprotected, the contractible flow of the spur dike changed the velocity distribution and the velocity around the spur dike increased. As the water was blocked by the spur dike, the downward flow formed. Figure 18b shows that when a permeable collar was used, the permeability of the collar reduced the velocity around the spur dike obviously, so the scour of the riverbed was reduced. When the porosity of the collar increased, the ability of the collar to block the downward flow decreased gradually. The protective effect was gradually weakened, and the local scour of the spur dike was intensified. It was because the permeability of the collar allowed part of the water to flow through the pores. The velocity of the flow field around the collar was reduced significantly by water flow in different directions. The local scour around the spur dike was reduced effectively. In the meantime, the upstream flow met at the collar and produced a circumferential flow. The speed of water in the collar was small, but the speed around the collar was high. Due to the high-speed flow around the spur dike, the local scour at the head of the spur dike was much worse.

Figure 18c shows that when the solid collar was used for protection, the collar's barrier to the downward flow was further enhanced. The intensity of the downdraft in front of the spur dike was greatly reduced. The horseshoe vortex above the collar was blocked by the collar, which reduced the scour of the riverbed. The flow field over the collar weakened the flow field around the spur dike to a certain extent. The velocity around the collar was smaller than that without protection, thus it reduced the local scour of the spur dike effectively.

**Figure 19:**
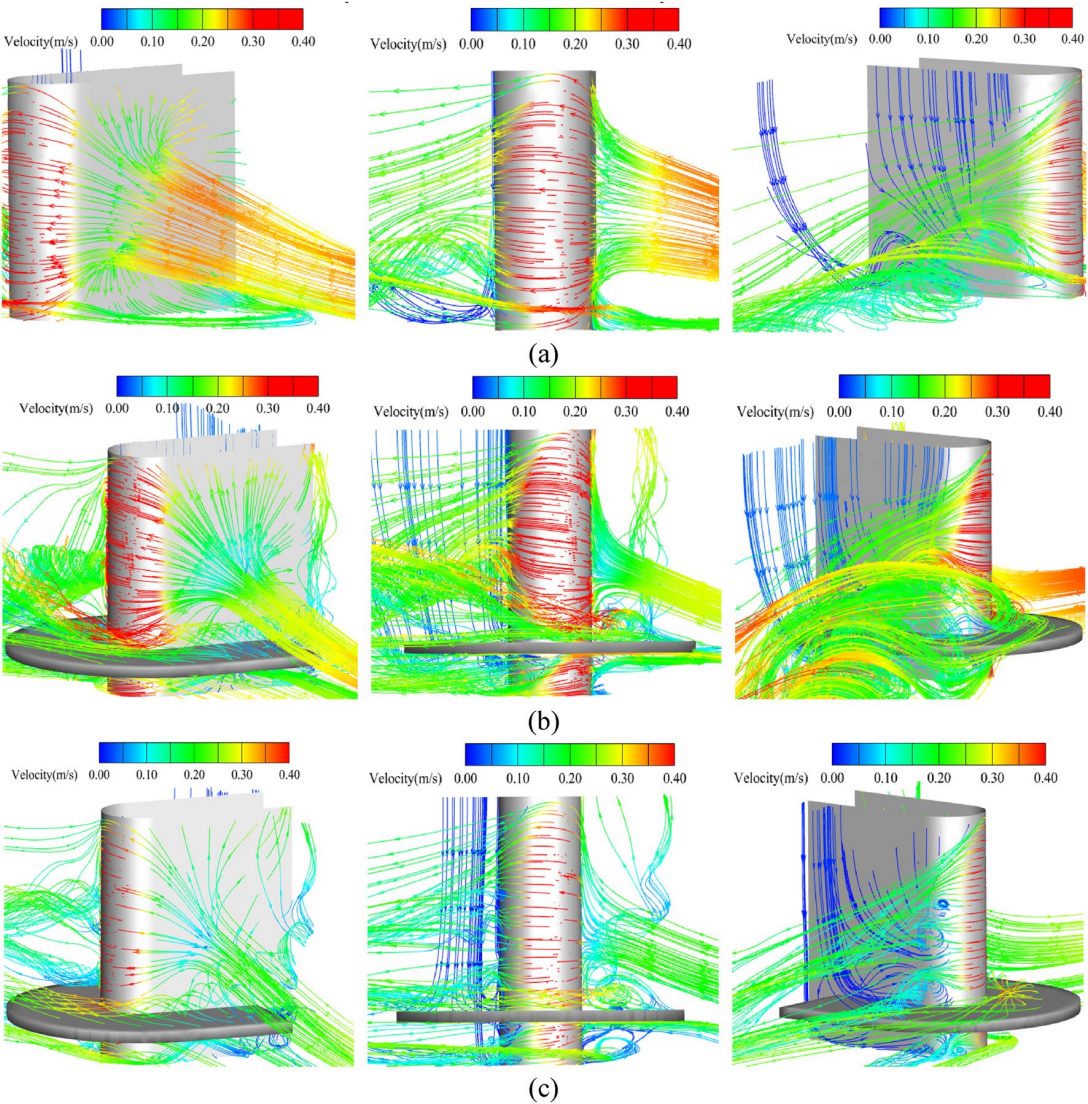
Streamlines around the spur dike (z = 0.005 m). (**a**) No.26 (without collar). (**b**) No.19 (L_C_/L = 0.6, L_K_/L = 0.5, γ = 0.7). (**c**) Test with the solid collar (L_C_/L = 0.6, L_K_/L = 0.5, γ = 0).

Figure 19 shows the streamline around the spur dike in No. 26 (without collar), No.19 (L_C_/L = 0.6, L_K_/L = 0.5, γ = 0.7), and the test with the solid collar (L_C_/L = 0.6, L_K_/L = 0.5, γ = 0).

Figure 19a shows that the upstream water flow was blocked by the spur dike and backwater was formed in front of the spur dike. Part of the water flowed downstream around the spur dike, and the other part of the water flowed to the bottom of the riverbed and became a descending flow. The flow of water and the side wall were separated from each other, which produced a vortex and resulted in a return flow area in front of the spur dike. The flow velocity around the spur dike was redistributed. The flow velocity near the spur dike increased obviously, and local scour was produced under the joint action of the horseshoe vortex. The water flowed around the spur dike and was separated into a vertical spiral vortex. The water flow inside the vortex presented complex three-dimensional characteristics. Due to the separation of the vortex at the spur dike head, the sediment was transported downstream.

Figure 19b and c show that when the collar was used for protection, the water flow formed a complex shape around the collar The water flow was divided by the collar into the above and flow parts. Some vortices formed on the surface of the collar, which inhibited the formation of vortices below the collar. The water flow was blocked by the collar, which greatly weakened the intensity of the downward flow in front of the spur dike. The local scour around the spur dike was mainly caused by the flow below the collar. However, when the collar was a solid structure, the collar had a stronger blocking effect on the downward flow than the permeable collar. The flow velocity of water under the collar decreased significantly. The smaller the porosity was, the smaller the flow velocity was, the vortex intensity was also decreased.

### Distribution of shear stress on the riverbed

**Figure 20:**
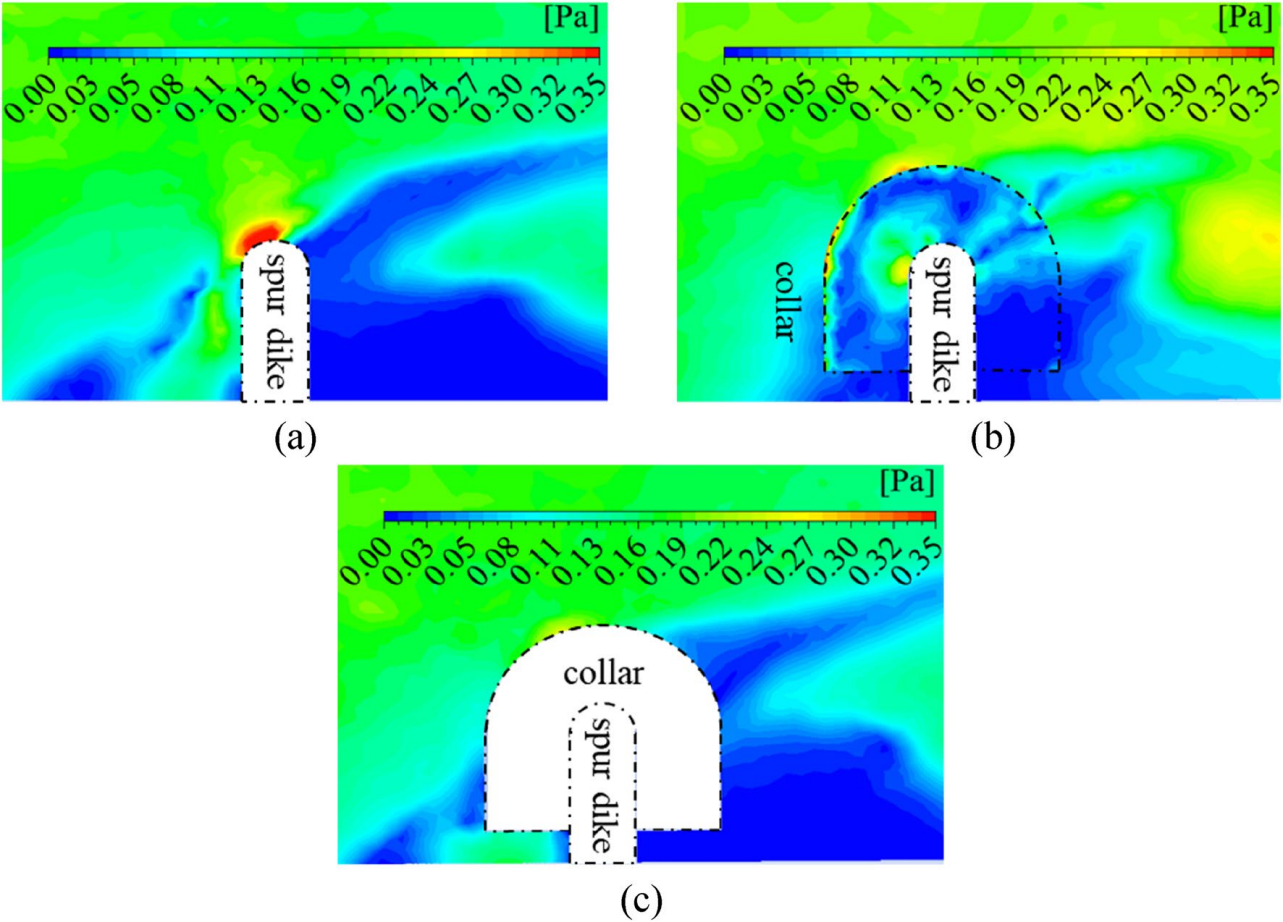
Shear stress distributions near the riverbed (z = 0.005 m). (**a**) No. 26 (without collar). (**b**) No. 19 (L_C_/L = 0.6, L_K_/L = 0.5, γ = 0.7). (**c**) Test with the solid collar (L_C_/L = 0.6, L_K_/L = 0.5, γ = 0).

The shear stress near the riverbed can reflect the intensity of local scour directly. Figure 20 shows the shear stress near the riverbed in the No. 26 group (without collar), No.19 (L_C_/L = 0.6, L_K_/L = 0.5, γ = 0.7), and tests with the solid collar (L_C_/L = 0.6, L_K_/L = 0.5, γ = 0). Figure 20a shows that when the spur dike was unprotected, the velocity around the spur dike increased significantly. A high-stress zone was formed in front of the spur dike. The flow velocity in front and behind the spur dike was small, and a low-stress zone was formed. Results in the study were consistent with the existing results.

Figure 20b shows that when the collar was used for protection, the permeability of the collar could reduce the flow velocity. The shear stress near the riverbed was significantly reduced and the local scour was reduced. The permeability and size of the collar had significant effects on the shear stress and distribution characteristics. When the porosity of the collar was small, the velocity around the collar and the shear stress near the riverbed were decreased obviously. As the porosity increased, the barrier effect of the collar on the falling flow was weakened, so that the shear stress and local scour near the riverbed increased significantly. The results are consistent with the test results in Fig. 8c. Figure 20c shows that when the collar was a solid structure, the water-blocking effect of the collar was increased significantly. The flow velocity and shear stress were reduced significantly. Local scour around the spur dike was reduced. Those results were consistent with the results of the test in Fig. 11.

## Conclusions

The effect of the length, width, and porosity of the collar on the local scour reduction around the spur dike was studied by model tests and numerical simulations in this study. The main conclusions are as follows:

According to the results of ANOVA, the length, width, and porosity of the collar can impact the local scour reduction around the spur dike significantly, and the width of the collar contributes the most to the reduction effect of the collar. The local scour reduction efficiency can reach up to 56.9% when a collar is used.

A formula for the local scour reduction efficiency of the collar is proposed based on the results of the flume test by regression analysis. The deviation between the value calculated by the formula and that of the experiment is within ± 4%.

When a collar is used, the flow is divided into two parts: the flow above the collar and below the collar. This can restrain the formation of the horseshoe vortex near the surface of the sediment. As the porosity is decreased, the ability of the collar to block the downward flow is increased, the flow velocity around the spur dike is reduced and the local scour reduction effect is improved.

When a collar is used, the shear stress near the riverbed is decreased significantly. When the width of the collar is increased, the shear stress near the riverbed decreases further, and the sediment accumulation behind the spur dike is intensified.

Compared to the case without collar, the flow velocities around the spur dike in cases with permeable collar and solid collar reduced by 45% and 25%, respectively, and the shear stresses reduced by 20% and 28.6%, respectively.

The permeability of the collar was considered in this study, and the results of the study can be used as a reference for determining the size of protection when using permeable materials such as gabions for local scour reduction.

In this study, the position of the collar was fixed, which had a significant effect on its long-term protective effect. New protection measures that are adaptive to the terrain can be built upon based on this study, and research on its characteristics of local scour reduction can be conducted in the future.

## Data Availability

The data that support the findings of this study are available from the corresponding author upon reasonable request.

## References

[1] Wu YX, Wang JJ, Yuan XM (2020). Research summary on scouring and protection safety of spur dike. J. Nat. Disasters.

[2] Radan P (2016). Flow and scour pattern around submerged and non-submerged T-shaped spur dikes in a 90° bend using the SSIIM model. Int. J. River Basin Manag..

[3] Mohammad V, Yaser S, Shaker SH (2016). Effects of distance between the T-shaped spur dikes on flow and scour patterns in 90° bend using the SSIIM model. Ain Shams Eng. J..

[4] Mehraein M, Ghodsian M, Mashizi KM (2017). Experimental study on flow pattern and scour hole dimensions around a T-shaped spur dike in a channel bend under emerged and submerged conditions. Int. J. Civil Eng..

[5] Maryam A, Mohammad V, YeeMeng C (2021). Effect of T-shaped spur dike length on mean flow characteristics along a 180-degree sharp bend. J. Hydrol. Hydromech..

[6] Li WY (2023). Study on the hydraulic characteristics of Ding dam under different permeable conditions. Dams Saf..

[7] Liu DD, Lv YC, Yang X (2023). Study on the influence of different dam spacing on water flow characteristics. Ningxia Eng. Technol..

[8] Wen WJ (2022). Model test study on the influence of Ding dam angle on the flow regime of curved water. Tech. Superv. Water Conserv..

[9] Zheng GY (2021). Scour Characteristics and Optimal Design of Permeable Gabion Groins.

[10] Han, M. J. Analysis of the scouring mode and water flow morphology of the Ding Dam. *Tech. Superv. Water Conserv.* (2):161–164, 227, 232. 10.3969/j.issn.1008-1305.2023.02.042 (2023).

[11] Vaghefi M, Ghodsian M, Akbari M (2017). Experimental investigation on 3D flow around a single T-shaped spur dike in a bend. Periodica Polytech. Civil Eng..

[12] Vaghefi M, Alavinezhad M, Akbari M (2016). The effect of submergence ratio on flow pattern around short T-head spur dike in a mild bend with rigid bed using numerical model. J. Chin. Inst. Eng..

[13] Mohammad V (2017). Effect of T-shaped spur dike on flow separation in a 90° bend using SSllM model. J. Natl. Sci. Found. Sri Lanka.

[14] Zhang HN, Jing HF (2023). Three-dimensional numerical simulation study of water flow movement under the influence of multi-stage Ding dam. Ningxia Eng. Technol..

[15] Li, G. J. & Zhong, L. Numerical simulation of local scouring characteristics of stepped Ding dam. *Water Transp. Eng.* (8), 93–100, 189. 10.3969/j.issn.1002-4972.2021.08.016 (2021).

[16] Ning J, Li GD, Ma M (2017). 3D numerical simulation for flow and local scour around spur dike. J. Hydrodyn..

[17] Hu ZY (2021). Three-dimensional numerical simulation study of the influence of Ding dam angle on the surrounding flow field. Eng. Technol. Res..

[18] Jourabi, Y. J., Ardeshir, A. & Karami, H. Experimental research on sacrificial spur dikes to reduce scouring. In *36th IAHR World Congress*, 4009–4017 (Iahr-Int Assoc Hydro-Environment Engineering Research, 2015).

[19] Huang W, Creed M, Chen F, Liu HH, Ma A (2018). Scour around submerged spur dikes with flexible mattress protection. J. Waterway Port Coast. Ocean Eng..

[20] Kumcu SY, Kokpinar MA, Gogus M (2021). Effect of collars on the downstream movement of the maximum scour depth location around bridge abutments and piers. Iran. J. Sci. Technol.-Trans. Civil Eng..

[21] Melville B, Ballegooy SV, Coleman S (2006). Countermeasure toe protection at spill-through abutments. J. Hydraul. Eng..

[22] Ding JJ, Lu Y, Lu YJ (2014). Hydrodynamic characteristics of a spur dike with permeable groyne head and its application. Hydro-Sci. Eng..

[23] Jiang XY (2010). Stability analysis of loose rock casting dam in Nanjiang River channel. Heilongjiang Sci. Technol. Water Conserv..

[24] Liu Y, Jiang EH, Li JH (2007). Layout of groins and relations with the purpose of river training. Yellow River.

[25] Tian WP, Li HP (2002). Influence on scour depth of spur dike parameter. J. Chang'an Univ. (Nat. Sci. Ed.).

[26] Zhang K (2017). Experimental Study on the Jetty Head Scour Law of Hydraulic Flashboard Permeable Spur Dike.

[27] Zhao K (2009). Experimental Study on Simulation of Local Scour at Bridge Abutments.

[28] Gao H, Wang QS (2022). Numerical study on the influence of riverbed median particle size on scour pit evolution process around bridge pier. J. Yangtze River Sci. Res. Inst..

[29] Dou XP, Wang XM, Lou B (2005). General scouring model test of dike′s head under tidal currents and waves. Hydro-Sci. Eng..

[30] Fang DX, Wang J (1992). Discussion on the calculation model of bed sand uplift flow rate and local maximum depth of scour at the head of spur dike. J. Sediment Res..

[31] Raudkivi AJ, Ettema R (1983). Clear-water scour at cylindrical piers. J. Hydraul. Eng..

[32] Fang SL, Chen H, Shi XF (2016). Experimental study on protection effect of flow-altering countermeasures against clear water scour at bridge piers. J. Chongqing Jiaotong Univ. (Nat. Sci.).

[33] Jahangirzadeh A, Basser H, Akib S (2014). Experimental and numerical investigation of the effect of different shapes of collars on the reduction of scour around a single bridge pier. PLoS ONE.

[34] Zhao M (2012). Experimental study of local scour around subsea caissons in steady currents. Coast. Eng..

[35] Kumar V, Raju KGR, Vittal N (1999). Reduction of local scour around bridge piers using slots and collars. J. Hydraul. Eng..

[36] Liu A, Tian L (2022). Influence of size of local ice jam on flow and scour around bridge piers. J. Highway Transp. Res. Dev..

[37] Ning J (2019). Numerical Simulation of Flow Field and Local Scour Around Spur Dike.

[38] Jiang Y (2020). Study on Parameter Optimization of Piano Key Weir Upside Down Ratio and Numerical Simulation of Downstream Scour.

